# Water at electrode–electrolyte interfaces: combining HOD vibrational spectra with *ab initio*-molecular dynamics simulations†

**DOI:** 10.1039/d4sc04766d

**Published:** 2024-09-23

**Authors:** Pavithra Gunasekaran, Xianglong Du, Andrew Burley, Jiabo Le, Jun Cheng, Angel Cuesta

**Affiliations:** a Advanced Centre for Energy and Sustainability (ACES), School of Natural and Computing Sciences, University of Aberdeen AB24 3UE Aberdeen Scotland UK angel.cuestaciscar@abdn.ac.uk; b State Key Laboratory of Physical Chemistry of Solid Surfaces, iChem, College of Chemistry and Chemical Engineering, Xiamen University Xiamen 361005 China; c Ningbo Institute of Materials Technology and Engineering, Chinese Academy of Sciences Ningbo Zhejiang 315201 China; d Centre for Energy Transition, University of Aberdeen AB24 3FX, Aberdeen Scotland UK

## Abstract

We have undertaken a vibrational study of the structure of interfacial water and its potential dependence using H_2_O : D_2_O mixtures to explore the O–H and O–D stretching modes of HOD as well as the bending modes of HOD and H_2_O. Due to the symmetry reduction, some of the complexity characteristic of the vibrational spectrum of water is removed in HOD. Coupled with potential-dependent *ab initio* simulations of the gold–water interface, this has enabled a deeper insight into the hydrogen-bond network of interfacial water and into how it is affected by the applied potential. Possibly the most important conclusions of our work are (i) the absence of any ice-like first layer of interfacial water at any potential and (ii) that interfacial water reorients around a stable backbone of hydrogen bonds roughly parallel to the electrode surface. At *E* > pzc, interfacial water molecules are oriented with the oxygen lone pairs towards the surface and form exclusively or nearly exclusively hydrogen-donating hydrogen bonds with other water molecules. At *E* < pzc, the oxygen lone pairs instead point away from the surface, but the population of hydrogen-donating water molecules does not vanish. In fact, the population of hydrogen-accepting water molecules only dominates at considerably negative charge densities, due to the weak interaction of the hydrogen atoms of interfacial water molecules with the Au surface.

## Abstract 2

ATR-SEIRAS and *ab initio* MD simulations reveal details of the hydrogen bond network of water at the electrode–electrolyte interface.

## Graphical abstract

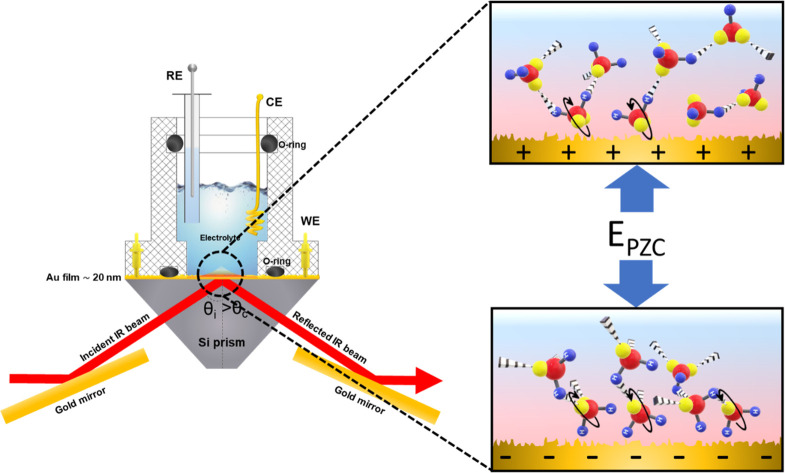

## Introduction

For a simple molecule, water displays a surprisingly rich variety of behaviours and unusual properties. It has unusually high melting and boiling points, an exceptionally high heat capacity and latent heats of fusion and vaporisation, and the highly unusual property of having lower density in its solid form, ice, than in its liquid form. The common reason for all these exceptional properties is the ability of water molecules to form hydrogen bonds with its neighbours. Typically, a water molecule can form four hydrogen bonds: two with its hydrogen atoms (conventionally termed as “donor” hydrogen bonds) and two with the lone pair electrons on its oxygen atom (“acceptor” hydrogen bonds). In ice, each molecule possesses four hydrogen bonds in a tetragonal configuration. In water, thermal agitation results in breaking some of these hydrogen bonds, so the average number of hydrogen bonds per water molecule is between 3.5 and 3.7.^1^

Vibrational spectroscopy has proven itself to be a useful tool to probe hydrogen bonding in liquid water, because the position and shape of the O–H stretching band and the H–O–H bending mode are highly sensitive to the hydrogen bond environment. In the gas phase, water has three vibrational bands: the symmetric stretch, *ν*_1_ at 3657 cm^−1^, the antisymmetric stretch, *ν*_2_ at 3756 cm^−1^ and the bending mode, *δ*_HOH_ at 1595 cm^−1^. On the contrary, in liquid water the complexity of the hydrogen bonding network underlying the shape of the O–H stretching band makes its interpretation difficult. Although the traditional interpretation is that the line shape arises from the contribution of different populations of water which experience different hydrogen bonding environments (mixture model), advances in the last 15 years suggest that an interpretation based on the delocalisation of instantaneous vibrational eigenstates over a substantial number of chromophores (continuum model) through coupling of the stretching modes of different molecules (*i.e.*, intermolecular intramode coupling), as well as on the Fermi resonance between the overtone of *δ*_HOH_ and the stretch mode (*i.e.*, intramolecular intermode coupling), is much more appropriate.^2–7^ In the liquid form of water, intra- and intermolecular coupling of *ν*_1_ and *ν*_2_ results in a broad *ν*_OH_ peak at around 3450 cm^−1^.^2^ This broad O–H stretching band typically shows a shoulder on its low frequency end due to a Fermi resonance between the first overtone of the bending band and the broadened symmetric stretch.^6,8,9^ Due to the symmetry reduction from point group *C*_2v_ to *C*_s_, some of this complexity is removed in the vibrational spectrum of HOD. Although the hydrogen-bond network remains, and so does, albeit altered, the delocalisation of the vibrational stretching eigenstates through intermolecular intramode coupling, now *ν*_OH_ and *ν*_OD_ are independent of each other, and the Fermi resonance between the first overtone of the *δ*_HOD_ band (appearing around 1450 cm^−1^) and either the *ν*_OH_ or *ν*_OD_ stretch becomes impossible. By diluting either H_2_O in D_2_O or D_2_O in H_2_O, solutions containing either only HOD and D_2_O or only HOD and H_2_O, respectively, can be prepared. Please note that, because the dissociation constant of D_2_O (p*K*_D_2_O_ ≈ 15) is smaller than that of H_2_O (p*K*_w_ ≈ 14),^10^ the equilibrium H_2_O + D_2_O ⇌ HDO is *K*_eq_ < 4,^11–14^ and an estimation of the concentrations of H_2_O, D_2_O and HOD in any H_2_O : D_2_O mixture based merely on a complete isotope scrambling will not yield the correct composition.^15^ For example, a 25% solution in H_2_O (D_2_O) and 75% in D_2_O (H_2_O) will be approximately 50% HOD and 50% D_2_O (H_2_O), with H_2_O (D_2_O) amounting to only a few percent mole fraction.^15^ Thus, the band in the *ν*_OH_ (*ν*_OD_) region can be attributed to HOD alone, while the band in the *ν*_OD_ (*ν*_OH_) region will contain contributions from both HOD and D_2_O (H_2_O). The *δ*_HOD_ band also appears clearly separated (around 1450 cm^−1^) from *δ*_HOH_ (around 1645 cm^−1^) and *δ*_DOD_ (around 1210 cm^−1^) and is an additional powerful probe of the degree of hydrogen bonding.^7^

If understanding the behaviour of bulk water is surprisingly difficult, given the simplicity of a rather symmetric triatomic molecule, interfaces impose an additional layer of complexity. Yet the properties of interfacial water are essential to the understanding of a large and varied set of phenomena, including, but not limited to, ice formation and growth, protein folding, and electrochemical interfaces and processes. Detection of spectroscopic signals from interfaces is a nontrivial problem, as they are easily obscured by the bulk response. Surface enhanced infrared absorption spectroscopy in attenuated total reflectance mode (ATR-SEIRAS) has proven itself to be an extremely powerful technique to characterise the electrode–electrolyte interface. Ataka *et al.*^9^ used ATR-SEIRAS to investigate the potential dependence of the structure of interfacial water using preferentially (111)-oriented gold electrodes in perchloric acid solutions as a model system. They concluded that water dipoles reorient around the potential of zero charge (pzc) in response to the surface charge changing from negative to positive. Evidence was also provided of the presence of hydronium and perchlorate at potentials negative and positive of, respectively, the pzc. An ice-like water bilayer was proposed to exist at potentials slightly positive of the pzc, supported by the presence of a weak band centred at approximately 3200 cm^−1^. This seminal work remains the keystone of our understanding of the potential-dependent structure of water at electrode–electrolyte interfaces.

Here we make use of H_2_O : D_2_O mixtures to build on Osawa's and co-workers seminal work.^9^ By exploring potential-induced changes in either the O–H stretching band of 1 : 3 H_2_O : D_2_O mixtures or in the O–D stretching band of 1 : 3 D_2_O : H_2_O mixtures and in the corresponding bending bands, we unveil information regarding changes in the orientation of interfacial water molecules and in the interfacial hydrogen-bond environment. We combine the vibrational information obtained from *in situ* ATR-SEIRA spectra of the interface between a Au electrode and 0.1 M HClO_4_ solutions in these H_2_O : D_2_O mixtures with potential-dependent *ab initio* molecular dynamics (PD-AIMD) simulations to obtain a deeper insight into the details of the structure of water at the electrode–electrolyte interface.

## Results

### H enrichment at the gold–electrolyte interface

**Fig. 1:**
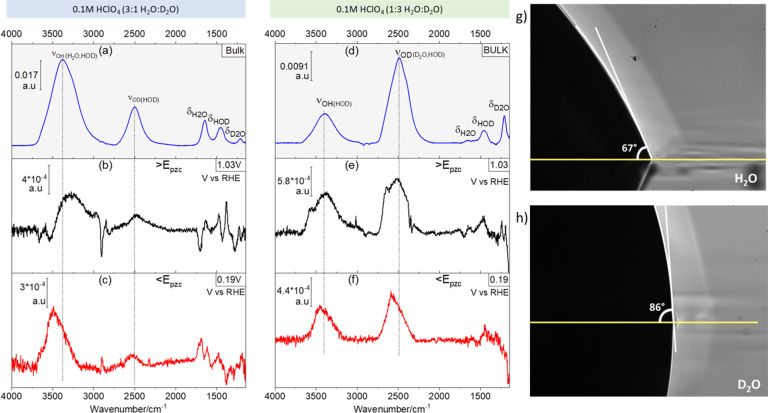
H enrichment at the Au–electrolyte interface. (a) ATR absorption spectrum of the bulk of a 0.1 M HClO_4_ solution in a H_2_O : D_2_O 3 : 1 mixture. (b and c) Characteristic ATR-SEIRA spectra of the interface between a Au electrode and a 0.1 M HClO_4_ solution in a H_2_O : D_2_O 3 : 1 mixture positive (b) and negative (c) of the pzc. (d) ATR absorption spectrum of the bulk of a 0.1 M HClO_4_ solution in a H_2_O : D_2_O 1 : 3 mixture. (e and f) Characteristic ATR-SEIRA spectra of the interface between a Au electrode and a 0.1 M HClO_4_ solution in a H_2_O : D_2_O 1 : 3 mixture positive (d) and negative (e) of the pzc. (g and h) Contact angles of a drop of H_2_O (g) and of D_2_O (h) on a thin Au film deposited on Si. The vertical lines in (a–f) highlight the higher frequency of *ν*_OH_ and *ν*_OH_ at the interface as compared to the bulk. ATR absorption spectra in (a) and (d) were obtained using the spectrum of an uncoated and dry Si prism as background. ATR-SEIRA spectra in (b), (c), (e) and (f) were obtained using the spectrum collected at 0.57 V as background.

Fig. 1 shows ATR absorption spectra of the bulk of 0.1 M HClO_4_ solutions in 3 : 1 (Fig. 1a) and 1 : 3 (Fig. 1d) H_2_O : D_2_O mixtures. Additionally, representative ATR-SEIRA spectra of water at the interface between the above-mentioned solutions and a gold electrode negative (Fig. 1b and e) and positive (Fig. 1c and f) of the pzc are shown. The O–H stretching bands (*ν*_OH_, around 3450 cm^−1^) are in all cases broader than their O–D counterpart (*ν*_OD_, around 2500 cm^−1^). This is due to the reduced anharmonicity of O–D bonds as compared with O–H bonds.^16,17^

The expected ratio of approximately 3 : 1 between the integrated intensities of *ν*_OH_ and *ν*_OD_ in favour of the majority component is found in the corresponding ATR absorption spectra of the solution bulk (see Fig. 1a and d). Quite surprisingly though, at the electrode–electrolyte interface the *ν*_OH_ band is always more intense than expected (see Fig. 1b–e): in 3 : 1 H_2_O : D_2_O mixtures the integrated intensity of the *ν*_OH_ band is *ca.* 5 times larger than that of *ν*_OD_, both positive and negative of the pzc (Fig. 1b and c, respectively), whereas in 1 : 3 H_2_O : D_2_O mixtures the integrated intensity of the *ν*_OD_ band is only *ca.* 1.2 times larger than that of *ν*_OH_ (Fig. 1e and f). This unexpected and, to the best of our knowledge, unreported enhancement of *ν*_OH_ over *ν*_OD_ at the electrical double layer, suggests an unexpected enrichment of H over D at the electrode–electrolyte interface. The existence of such an enrichment in H is confirmed by the difference between the contact angle of a drop of H_2_O (Fig. 1g) and that of a drop of D_2_O (Fig. 1h) on Au: the contact angle is *ca.* 23% lower for H_2_O (66.50° ± 0.87) than for D_2_O (86.67° ± 0.58), implying a higher interfacial tension at the Au–D_2_O interface (*γ*_D_2_O_) than at the Au–H_2_O interface (*γ*_H_2_O_). Because the interfacial tension corresponds to the free energy of formation of the interface, a higher *γ*_D_2_O_ should result in an enrichment of the interface in H over D in H_2_O/D_2_O mixtures, as in fact revealed by the ATR-SEIRA spectra of the Au–electrolyte interface. This difference between *γ*_D_2_O_ and *γ*_H_2_O_ can be explained by the subtle changes in the oxygen–hydrogen bonds brought about by substituting D for H. The O–D bond is 3% shorter than the O–H bond,^1^ and D_2_O has a slightly larger dipole moment than H_2_O.^18,19^ Accordingly, the surface tension (*i.e.*, the interfacial tension of the liquid–air interface) of D_2_O is 6.8% lower than that of H_2_O.^20^ Arguably, the 23% difference in the contact angles reported in Fig. 1g and h is much larger than expected if the differences between *γ*_D_2_O_ and *γ*_H_2_O_ are of the same magnitude. However, our observations suggest an opposite trend (*i.e.*, *γ*_D_2_O_ > *γ*_H_2_O_) and a difference between interfacial tensions > 6.8% cannot be excluded. It is also very possible that the lower purity of bottled D_2_O, as opposed to the ultrapure H_2_O from a water purification system, enhances the differences in contact angle. In support of our proposed enrichment in H_2_O at the Au–water interface, enrichment in D_2_O has been observed at very hydrophilic interfaces immersed in H_2_O : D_2_O mixtures.^21^ Enrichment in H_2_O at much less hydrophilic metallic surfaces seems therefore plausible.

Also evident from comparing Fig. 1a with Fig. 1b and c, and Fig. 1d with Fig. 1e and f, respectively, is a blue shift at the interface of the *ν*_OD_ and *ν*_OH_ bands of HOD, particularly evident negative of the pzc (Fig. 1c), as well as a slight red shift of *δ*_HOH_ and *δ*_HOD_ (the overlap of the *δ*_DOD_ around 1200 cm^−1^ band with the intense and broad Si–O band of silicon dioxide^22^ prevents a proper analysis of *δ*_DOD_). These shifts are consistent with a lower degree of hydrogen bonding in interfacial water, as compared to the bulk, and were indeed to be expected, as the presence of the electrode surface prevents formation of hydrogen bonds with non-existing water molecules at the electrode side of the interface.

### Potential dependence of the ATR-SEIRA spectra of interfacial HOD

Due to the very intense electric fields (≈10^9^ V m^−1^) at the electrode–electrolyte interface, the average orientation of interfacial water dipoles should be expected to be affected by the electrode potential. Such electric field-induced ordering of interfacial water, with the negative end of the water dipole pointing either towards or away from the electrode surface positive and negative, respectively, of the pzc, is actually an essential part of classical models of electrochemical interfaces.^23–26^ Similarly, both experimental evidence^9^ and computational simulations^27–30^ suggest that, at or around the pzc, the average orientation of water dipoles is parallel to the electrode surface.

**Fig. 2:**
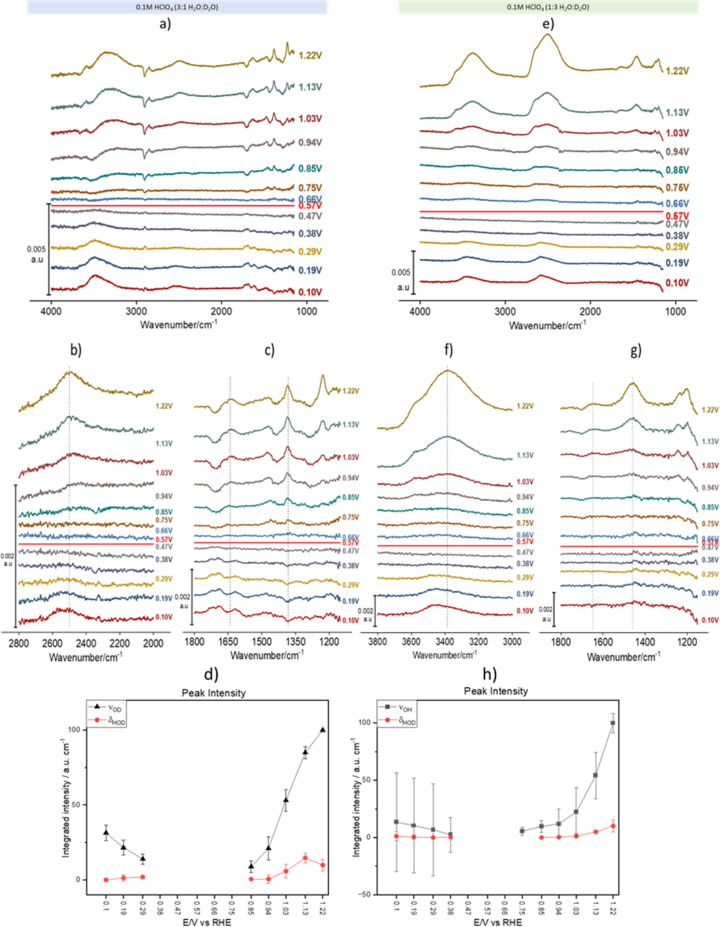
Potential-dependent ATR-SEIRA spectra of the Au-electrolyte interface. (a–c and e–g) ATR-SEIRA spectral series of the interface between a thin-film Au electrode deposited on Si and a 0.1 M HClO_4_ solution in H_2_O : D_2_O 3 : 1 (a–c) and in H_2_O : D_2_O 1 : 3 (e–g). (a) and (e) present the whole available spectral range, whereas (b) and (f) focus on the O–D and O–H spectral region, respectively, and (c) and (g) on the HOD bending region. The vertical blue lines are just a guide to the eye to help identify frequency shifts positive and negative of the potential at which the background was collected (0.57 V *vs.* RHE, which corresponds approximately to the pzc). Spectra were collected during a linear potential sweep from 0.10 to 1.22 V *vs.* RHE at 5 × 10^−3^ V s^−1^. A background spectrum was collected at 0.10 V, but the spectra have been recalculated using the spectrum at 0.57 V as background. (d and h) Potential dependence of the integrated intensity of *ν*_OD_(HOD) (d, black symbols), *ν*_OH_(HOD) (h, black symbols) and of *δ*_HOD_ (red symbols in (d) and (h)). The vertical lines in (b), (c), (f) and (g) highlight the frequency shift of *ν*_OD_(HOD), *ν*_OH_(HOD), *δ*_HOD_ and *δ*_HOH_ when crossing the pzc. All spectra were calculated using the spectrum at 0.57 V as background.

Fig. 2 shows spectral series recorded during a linear potential sweep between 0.10 and 1.22 V *vs.* RHE at 5 × 10^−3^ V s^−1^. Although the background was recorded at 0.1 V, the spectra were recalculated using the spectrum at 0.57 V as background. This potential is very close to the pzc of Au(111) (0.475 V *vs.* SHE,^31^*i.e.*, 0.535 V *vs.* RHE in 0.1 M HClO_4_). It must be noted, however, that ours is a polycrystalline surface composed of facets exposing different crystallographic orientations and its pzc is ill defined, as each of those orientations has its own distinct pzc. At the overall pzc there will therefore be positively and negatively charged areas on the electrode surface. Hence, changes in the water structure in this region can be expected to be gradual and, in fact, the calculated spectra barely change when a spectrum collected at any potential between 0.47 and 0.57 V is used as background (see the negligible intensity of the bands in this potential region, Fig. 2a–g). Fig. 2a–d and e–h correspond to experiments in 3 : 1 and 1 : 3 H_2_O : D_2_O mixtures, respectively. In Fig. 2a, the *ν*_OH_ band at wavenumbers > 3000 cm^−1^ contains contributions from both H_2_O and HOD (we will therefore from now on identify this band as *ν*_OH_(H_2_O, HOD)), while in Fig. 2e that band corresponds exclusively to *ν*_OH_ of HOD (*ν*_OH_(HOD)). Similarly, in Fig. 2e the *ν*_OD_ band between 2300 and 2800 cm^−1^ contains contributions from both D_2_O and HOD (*ν*_OD_(D_2_O, HOD)), whereas in Fig. 2a that band corresponds exclusively to *ν*_OD_ of HOD (*ν*_OD_(HOD)). In the bending region, Fig. 2a and c only show bands corresponding to *δ*_HOH_ (around 1650 cm^−1^) and *δ*_HOD_ (around 1450 cm^−1^), while Fig. 2e and g only show bands corresponding to *δ*_HOD_ (around 1450 cm^−1^) and *δ*_DOD_ (around 1200 cm^−1^), the latter strongly distorted by the Si–O stretching band of silicon dioxide. The absence of *δ*_DOD_ and *δ*_HOH_ in Fig. 2a, c, e and g, respectively, is evidence that the concentrations of D_2_O and H_2_O in 3 : 1 and 1 : 3 H_2_O : D_2_O mixtures, respectively, are negligible at the interface.

As expected, other than the narrower bandwidth of *ν*_OD_(D_2_O, HOD), the potential dependence of *ν*_OH_(H_2_O, HOD)) and *ν*_OD_(D_2_O, HOD) is similar to that reported by Ataka *et al.*,^9^ and will not be discussed. Some deeper insight into the structure of interfacial water and its dependence on the electrode potential can be gathered though from analysing the potential dependence of *ν*_OH_(HOD), *ν*_OD_(HOD), *δ*_HOH_ and *δ*_HOD_ (*δ*_DOD_ analysis is prevented by its overlap with the Si–O band of silicon dioxide).

*ν*_OD_(HOD) (Fig. 2b) and *ν*_OH_(HOD) (Fig. 2f) have very similar band shapes negative and positive of the pzc. The only differences are: (i) a lower peak frequency positive of the pzc and (ii) a more symmetric shape positive of the pzc, due to the vanishing of the long tail present at the low frequency side of the band negative of the pzc. This long low-frequency tail at *E* < pzc can be attributed to the interaction of either the D (Fig. 2b) or the H (Fig. 2f) atom of HOD with the metal surface (hydrogen bonding of water to metal surfaces can redshift the O–H stretching frequency below 3000 cm^−1^),^32^ which becomes less plausible or even impossible upon reorientation positive of the pzc. Recently, Li *et al.*^33^ have combined shell-isolated nanoparticle-enhanced Raman scattering (SHINERS) with PD-AIMD at potentials very negative of the pzc, and showed that, in this potential regime, the *ν*_OH_ frequency decreases significantly with increasing negatively potential. At this point the first water layer adopts a ‘two-H-down’ structure. These results are consistent with our attribution of the low-frequency tailing of the *ν*_OH_(HOD) and *ν*_OD_(HOD) bands to the weak interaction of either D or H with the gold surface.

The redshift of *ν*_OD_(HOD) (Fig. 2b) and *ν*_OH_(HOD) (Fig. 2f) at *E* > pzc suggests an increase in hydrogen bonding within interfacial water, either in number of hydrogen bonds per water molecules or in the strength of those hydrogen bonds (O–H bonds of H-donating water molecules are more strongly affected by hydrogen bonding than those of H-accepting ones^34^). The blue shift of both the *δ*_HOH_ and *δ*_HOD_ positive of the pzc (Fig. 2c and g) also suggests an increase in hydrogen bonding within the interfacial water. We suggest here that it is the increase in the strength of hydrogen bonding due to water reorienting with H up at *E* > pzc, and not the number of hydrogen bonds formed, which leads to these spectral changes, which is supported by the results of the AIMD simulations shown below.

The potential dependence of the integrated intensity of *ν*_OD_(HOD) and *ν*_OH_(HOD) (black symbols in Fig. 2d and h, respectively), as well as that of *δ*_HOD_ (red symbols in Fig. 2d and h) is similar to that reported by Ataka *et al.*^9^ for the intensity of *δ*_HOH_, which confirms that the observed spectral changes in all these bands are due to the potential-induced reorientation of interfacial water around the pzc.

A shoulder around 3570 cm^−1^ in the *ν*_OH_(HOD) and *ν*_OD_(D_2_O, HOD) bands, as well as a shoulder around 2790 cm^−1^ in *ν*_OD_(HOD) and *ν*_OD_(D_2_O, HOD)), appear around 0.94 V and increase with increasingly positive potential (Fig. 2a, b, e and f). A similar band was observed by Ataka *et al.*,^9^ who assigned it to water with a low degree of hydrogen bonding due to the disruption of the hydrogen-bond network by perchlorate ions (a known chaotrope^35,36^) accumulating in the electrical double layer at *E* > pzc. Without disagreeing with this assignment, based on the AIMD simulations described below, we can more specifically assign this band to the O–H stretching of water in very near proximity to perchlorate, *i.e.*, in its solvation shell. The high *ν*_OH_/*ν*_OD_ frequency in the solvation shell arises from the fact that ClO_4_^−^ does not form directed hydrogen bonds with water.^35^ In fact, as mentioned by Ataka *et al.*^9^ and shown in Fig. S1,† this high-frequency component of *ν*_OH_ of interfacial water is not observed in sulphuric acid solutions, as expected since SO_4_^2−^ does form directed hydrogen bonds with water.^36^

### Potential-dependent AIMD simulations

Computational simulations can provide a wealth of information regarding the microscopic structure of metal–water interfaces. An important problem, though, is the inclusion in those computational methods of the electrode potential, which is absolutely necessary for the simulations to accurately reflect reality and to enable comparison with experimental work.^37^ Recently, Le *et al.*^27^ have adapted the computational SHE method derived by Cheng and Sprik^38,39^ to calculate electrode potentials in AIMD simulations, and used it to calculate the pzc of Pt(111)–, Au(111)–, Pd(111)– and Ag(111)–water interfaces. More recently,^40^ we have also shown that electrode potentials at charge densities other than zero can also be calculated using that method if one or several ions (anions for positive and cations for negative electrode charge densities, respectively) are introduced in the electrolyte within the simulation cell. This approach leads to the emergence on the electrode surface of a charge identical in magnitude but opposite in sign to that corresponding to the sum of the ions present in the electrolyte. Details on how the potential in the SHE scale was calculated in our AIMD simulations are provided in the ESI.†

**Fig. 3:**
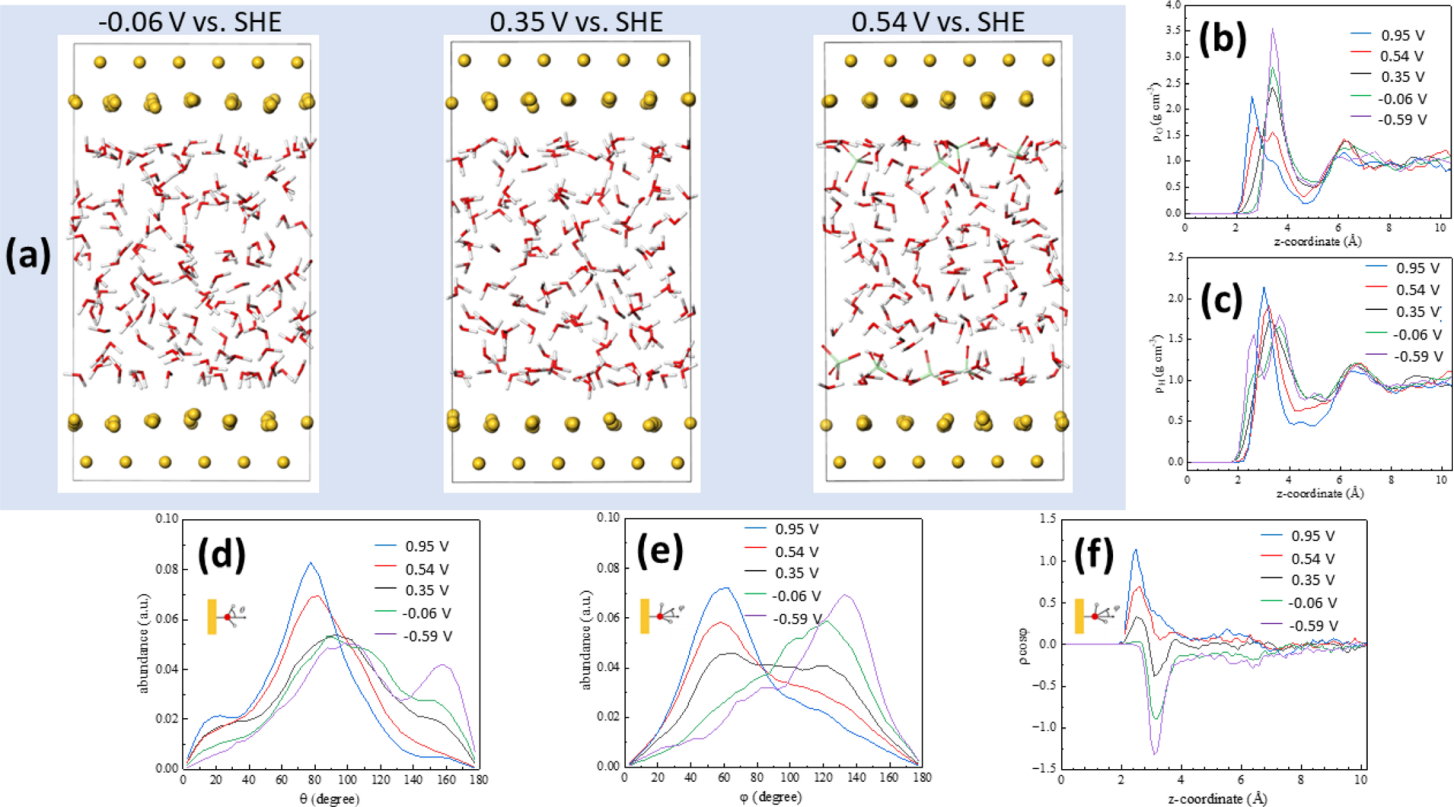
*Ab initio* molecular dynamics: potential-induced reorientation of interfacial water dipoles. (a) Snapshots of the Au(111)–water interface model at −0.06, 0.35 and 0.54 V *vs.* SHE, corresponding to electrode charge densities of −11.9, 0 (*i.e.*, the pzc) and +11.9 μC cm^−2^. (b and c) Oxygen (b) and hydrogen (c) density distributions at 0.95 (blue line), 0.54 (red line), 0.35 (black line), −0.06 (green line) and −0.59 (purple line) V *vs.* SHE. (d–f) Distribution of the angle between the O–H bond and the surface normal (d), of the water bisector and the surface normal (e) and of the product between the latter and the water density distribution (f). *z* = 0 corresponds to the position of the metal surface.

Fig. 3a shows snapshots of the Au(111)–water interface model at −0.06, 0.35 (the pzc) and 0.54 V *vs.* SHE. Fig. 3b and c show oxygen (*ρ*_o_) and hydrogen (*ρ*_H_) density distributions, respectively, as averaged from DFT-MD trajectories (shown in Fig. S2†) at +23.8, +11.9, 0, −11.9 and −23.8 μC cm^−2^, corresponding to 0.95, 0.54, 0.35, −0.06 and −0.59 V *vs.* SHE, respectively. The result at 0.35 V is consistent with the water density distribution profile at the pzc recently reported by us,^29^ with a single maximum at *z* < 4 Å, corresponding to the surface water layer. The fact that, as shown in Fig. 3b and c, the *ρ*_o_ and *ρ*_H_ maxima split into two above and below, respectively, the pzc, and that, as shown in Fig. 3d–f, at the pzc the surface water layer contains more or less identical populations of water molecules with the negative end of their electric dipole pointing towards and away of the electrode surface (just above 2 and around 3 Å from the surface, respectively, see Fig. 3e and f), suggests that this water surface layer is actually a bilayer composed of two parts with opposite net orientation dipoles.^29^ As noted in ref. 29, this is not the ice-like honeycomb bilayer structure, though. We identify the two components of this bilayer as water A (watA, closer to the electrode surface) and water B (watB, further away from the electrode surface). A smaller maximum at *z* > 6 Å, identified as watC, corresponds to water molecules barely affected by the metal surface, with no obvious net orientation and therefore similar to bulk water.^29^

Positive of the pzc (*i.e.*, at 0.54 and 0.95 V), the maximum in *ρ*_o_ observed at 0.35 V decreases in intensity, and a new maximum appears closer to the surface, whereas negative of the pzc (*i.e.*, at −0.06 and −0.59 V), the maximum in *ρ*_o_ observed at 0.35 V increases in intensity and no new maximum appears (Fig. 3b). This behaviour is mirrored by that of *ρ*_H_, which shows a new maximum emerging close to the Au(111) surface negative of the pzc while no new peak appears at 0.54 and 0.95 V (Fig. 3c). This is consistent with the potential-induced water reorientation deduced from the ATR-SEIRA spectra, but more details regarding the structural changes associated to such reorientation can be gathered by analysing the potential-induced changes in the angle between the O–H bond and the surface normal (Fig. 3d) and in the angle between the water bisector and the surface normal (Fig. 3e).

At the pzc (black lines in Fig. 3d and e), the majority of water molecules have one of their O–H bonds parallel to the electrode surface (maximum at *ca.* 90° in Fig. 3d), and there are approximately equal populations of water molecules with the other O–H bond pointing towards the surface (maximum around 155° in Fig. 3d) and away from the surface (maximum around 30° in Fig. 3d). This results in a broad peak centred around 90° in the distribution of the angle between the surface normal and the water bisector, with two small maxima around 60° and 120° (Fig. 3e, black line).

The population with one O–H bond pointing towards the surface essentially disappears at 0.54 and 0.95 V, but only at 0.95 V does this result in a slight increase of the population with one O–H bond pointing clearly away from the surface (maximum at 30° in Fig. 3d). Instead, it appears that most of the O–H bonds pointing towards the surface at the pzc reorient nearly parallel to, but slightly tilted away from, the surface: the maximum at *ca.* 90° at the pzc increases in intensity and shifts to *ca.* 80° and *ca.* 75° at 0.54 and 0.95 V, respectively (Fig. 3d). This is accompanied by a significant decrease in the population with an angle of *ca.* 120° between the molecule bisector and the surface normal and a similar increase in the population with an angle of *ca* 50° between the molecule bisector and the surface normal (Fig. 3e).

When potentials negative of the pzc are applied, it is the population with one O–H bond pointing away which nearly vanishes (see green and purple lines in Fig. 3d), but this time the number of O–H bonds parallel to the surface remains constant whereas the population with an angle between the O–H bond and the surface normal around 155° increases. In parallel, the population with an angle between the water bisector and the surface normal of *ca.* 50° decreases at the expense of the population at *ca.* 120° (Fig. 1e). Both positive and negative of the pzc, the population of water molecules with one O–H bond parallel to the electrode surface remains essentially the same as at the pzc (Fig. 3d).

**Fig. 4:**
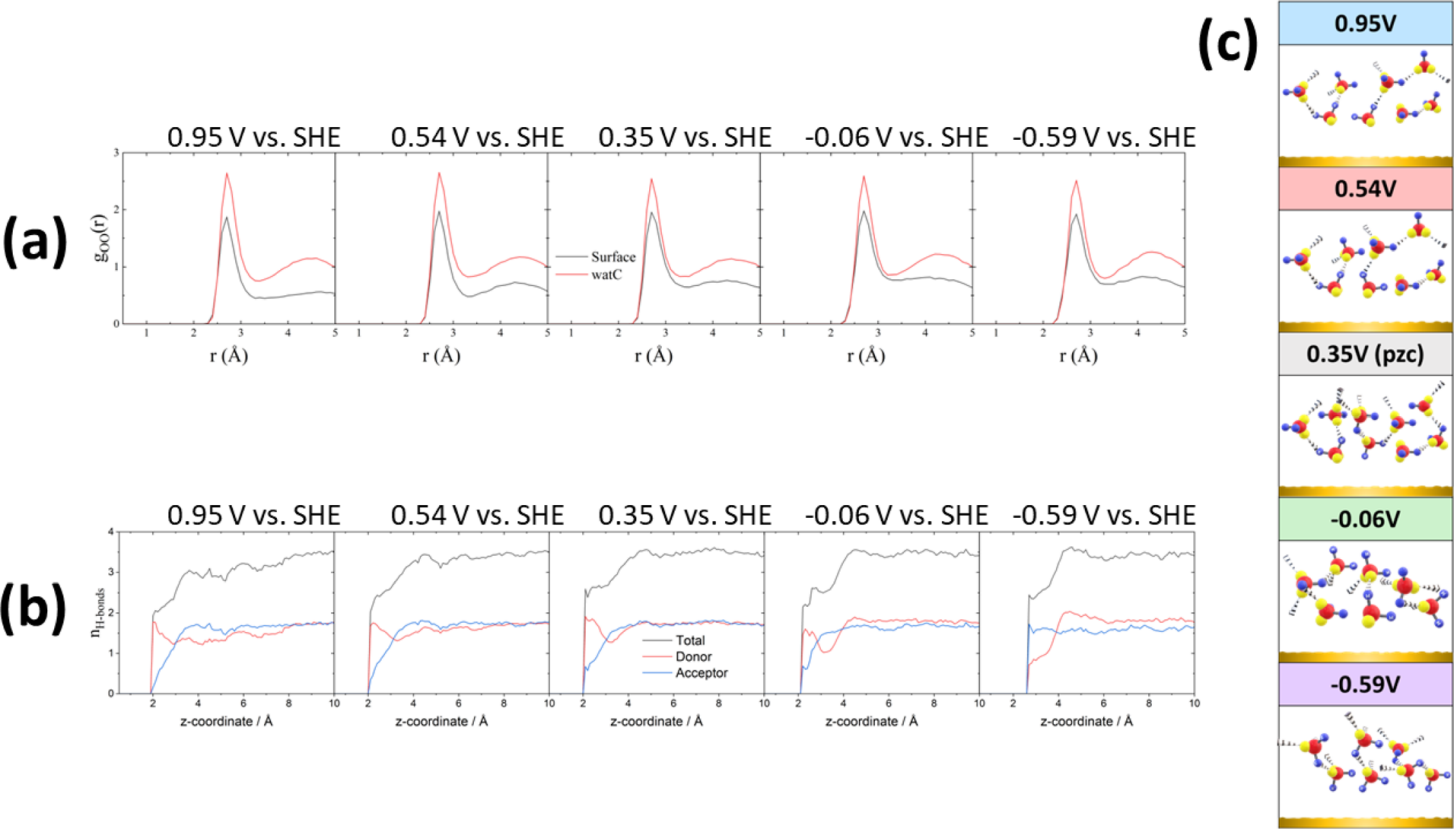
*Ab initio* molecular dynamics: hydrogen-bond network of interfacial water. (a) Water O–O radial distribution functions at 0.95, 0.54, 0.35, −0.06 and −0.59 V *vs.* SHE. Black and red lines correspond to the *g*_OO_ of surface water and watC (*i.e.*, bulk water), respectively. (b) Average numbers of water–water hydrogen bonds at 0.95, 0.54, 0.35, −0.06 and −0.59 V *vs.* SHE. A hydrogen bond is assumed to exist between two water molecules if the O–O distance is shorter than 3.5 Å and the O–O–H angle is less than 35°. Donor (red) means the water molecules donate a H atom when forming a hydrogen bond, while acceptor (blue) means they accept a H atom through one of the lone pairs on their oxygen atom. The black curve represents the total number of hydrogen bonds (sum of donor + acceptor). *z* = 0 corresponds to the position of the metal surface. (c) Cartoons showing a qualitative description of the interfacial water structure at each of the five calculated potentials.

All these potential-induced changes in the orientation of the O–H bonds and the bisector of the interfacial water molecules are consistent with the spectra presented in the preceding section. It is however particularly interesting to analyse the effect of such changes on the hydrogen-bond network at the interface. Fig. 4a shows plots of the O–O radial distribution functions (*g*_OO_(*r*)), which provides information regarding the solvation shell around each water molecule, positive of (0.95 and 0.54 V *vs.* SHE), negative of (−0.06 and −0.59 V *vs.* SHE) and at the pzc (0.35 V *vs.* SHE). There is a clear difference between the *g*_OO_(*r*) of surface water and of watC at all computed potentials. In all cases, the first *g*_OO_(*r*) peak of surface water shows a significantly lower intensity than that of watC, but it appears at roughly the same value of *r*, *i.e.*, the coordination number of surface water is lower, but the O–O distance does not change. This suggests that the presence of the metal surface partially strips water from the first solvation layer, but the interaction between water and the metal surface does not apparently affect the degree of hydrogen bonding with the remaining solvation layer, which is consistent with the experimental results in the preceding section. Analysis of the *g*_OO_(*r*) function allows us to estimate the coordination number of water (*i.e.*, the average number of hydrogen bonds per water molecule), which is found to be 3.6 for watC at all computed potentials except 0.95 V, when it is 3.4. In contrast, the coordination number for surface water is not only clearly lower than for watC and bulk water, but it shows clear potential dependence: it is 2.9 at and negative of the pzc but decreases to 2.8 and 2.5 at 0.54 and 0.95 V *vs.* SHE, respectively. The difference between the coordination number of surface water and that of bulk water is obviously due to the lack of water molecules on the electrode side of the interface, which decreases by just below one the number of hydrogen bonds any surface water molecule can form, and is consistent with previous work.^41–43^ Similarly, the decrease in the coordination number when crossing the pzc in the positive direction, which, as far as we know, had never been reported before, must obviously be due to the potential-induced reorientation of interfacial water.

These changes, particularly the decrease in the coordination number positive of the pzc, can be better understood by analysing the potential-dependent number of hydrogen-accepting and hydrogen-donating hydrogen bonds per molecule, as presented in Fig. 4b. As expected, the molecules at distances > 4 Å from the surface show a number of hydrogen bonds per molecule (3.6) characteristic of bulk water. The number of hydrogen bonds per molecule decreases quite sharply closer to the surface but remains clearly above 2 at and negative of the pzc. On the contrary, at 0.54 and, particularly, 0.95 V, the number of hydrogen bonds of those water molecules closest to the Au surfaces has decreased to nearly 2, and the number of hydrogen-accepting hydrogen bonds has decreased to nearly zero. This suggests that, at these potentials, the degree of orientation of the water molecules closest to the surface is high enough to prevent the lone pairs on their oxygen atoms from forming hydrogen bonds with other water molecules and explains the low coordination number resulting from Fig. 4a. In fact, positive of and at the pzc, and even at −0.06 V, the number of hydrogen-donating hydrogen bonds exceeds that of hydrogen accepting bonds, and only very negative of the pzc (−0.59 V *vs.* SHE) is the degree of orientation of the water dipoles high enough as to invert the population, resulting in approximately twice as many hydrogen-accepting as donating hydrogen bonds (Fig. 4b). This is in good agreement with our recent conclusion^27^ that even at the pzc water shares some electronic density with the gold surface, this being the main contributor to the difference between the work function and the pzc of Au(111). It is also in agreement with the weak interaction of the hydrogen atoms of interfacial water with the gold surface proposed in the previous section, to which we have attributed the tailing on the low-frequency side of the O–H and O–D stretching modes of HOD negative of the pzc, whereas a stronger hydrogen bond with the metal surface would appear below 3000 cm^−1^ and was not observed. Due to this weak interaction of the hydrogen atoms of interfacial water with the Au electrode, a very intense electric field and therefore a potential very negative with respect to the pzc is necessary to force the hydrogen atoms sufficiently far from the bulk of the electrolyte as to result in the number of hydrogen-accepting hydrogen bonds being higher than that of the hydrogen-donating bonds.

We compare now the structural analysis above with the results of Le *et al.*,^44^ who used the same method to simulate the potential-dependent structure of water at the interface between Pt(111) and an aqueous electrolyte containing Na^+^ and F^−^. We expect the effect of the applied potential on the structure of interfacial water to be similar in different metal–water interfaces due to the similar charge distribution at similar charge densities. Indeed, for the density distribution of water, the results in ref. 44 show that water molecules are closer to the metal surface at *E* < pzc, which is consistent with our simulations. As for the orientation distribution of water, our results also agree with ref. 44, as does the potential-induced change in the number of donor and acceptor hydrogen bonds. Such good agreement reinforces the reliability of the simulation results reported here.

To finalise this section, we have calculated the vibrational density of states (VDOS) of water near the interface at all five computed electrode potentials. The results are shown in Fig. S3,† in which we have also included the VDOS of a single water molecule around ClO_4_^−^. These calculations confirm that the shoulders at 3570 and 2790 cm^−1^ in the *ν*_OH_(HOD) and *ν*_OD_(HOD) bands, respectively, reported in the preceding section to appear around 0.94 V (Fig. 1a, b, e and f) correspond to water molecules around ClO_4_^−^, *i.e.*, in its solvation shell.

## Discussion

Most of our understanding of the structure of water at model electrode–electrolyte interfaces stems from the seminal work of Toney *et al.*^45,46^ and Ataka *et al.*^9^ Toney *et al.*^45,46^ were probably the first to use surface X-ray scattering (SXS) *in situ* to explore the structure of the electrode–electrolyte interface, specifically, that of a Ag(111) electrode in 0.1 M NaF. Their work revealed the expected reorientation of water from oxygen-up to oxygen-down when crossing the pzc in the positive direction and an increased density of water next to the surface, as compared to the bulk, in good agreement with the results presented by us here for the Au–electrolyte interface. They also argued that their results were inconsistent with an ice-like first layer of interfacial water.

Ataka *et al.*‘s^9^ conclusions were consistent with those of Toney *et al.*^45,46^ except for one thing: the presence of ice-like water in the double layer region of gold close to the pzc, which they inferred from the appearance of an absorption band around 3200 cm^−1^. In this work, no band can be identified in the *ν*_OD_ and *ν*_OH_ bands of HOD suggestive of the formation of an ice-like water layer at the interface. On the contrary, the shape of these bands is very similar positive and negative of the pzc, except for the long tailing at the low-frequency side at *E* < pzc that we have attributed to the weak interaction of the H or D atoms of HOD with the Au surface, possible only when water reorients with the negative end of its dipole pointing away from the electrode surface. In other words, in agreement with Toney *et al.*,^45,46^ we see no evidence that water is organised in an ice-like fashion at the interface. The difference between Ataka *et al.*’s^9^ and our conclusion, despite those conclusions being in both cases based on IR vibrational spectra, can be explained because, by using H_2_O : D_2_O mixtures, we have been able to analyse independently the O–D and O–H stretching modes of HOD, while Ataka *et al.*^9^ based their conclusions on the analysis of the O–H stretching of H_2_O. As explained above, the latter is strongly affected by inter- and intramode couplings, both within a molecule and between water molecules, which are strongly reduced in HOD due to the reduction of the molecule's symmetry. Changes in those intra- and intermolecular, intra- and intermode, couplings due to changes in the structure of interfacial water upon its potential-induced reorientation can easily lead to broadening and to shifting the peak frequency of the OH band of H_2_O and could easily be mistaken by the formation of an ice-like surface water layer. On the contrary, by reducing the degree of those vibrational couplings, the changes in the spectral shape of the O–H and O–D stretching modes are much smaller and have allowed us to clearly exclude the formation at room temperature of an ice-like surface water layer. It would be interesting, nonetheless, to expand our work and explore if such an ice-like water layer forms at any potential at lower temperatures.

The higher frequency of the *ν*_OD_ and *ν*_OH_ bands of interfacial water in our vibrational spectra, as compared with the solution bulk, suggests a decrease in the degree of hydrogen bonding in interfacial water, which is confirmed by our simulations and was indeed to be expected, because interfacial water molecules can only form hydrogen bonds to waters on the electrolyte side of the interface. Both the decrease in the frequency of *ν*_OD_ and *ν*_OH_ and the increase in the frequency of *δ*_HOH_ and *δ*_HOD_ with increasingly positive potentials are consistent with stronger hydrogen bonding positive of the pzc. However, the small magnitude of these frequency changes suggests that, rather than the number of hydrogen bonds per molecule, it is the type of hydrogen bond which is changing. This is confirmed by our PD-AIMD simulations, which show interfacial molecules acting nearly exclusively as hydrogen donors at *E* > pzc and increasingly as hydrogen acceptors at *E* < pzc. In fact, at −0.59 V the population of hydrogen acceptor water molecules exceeds that of hydrogen donors at the interface. Hydrogen accepting hydrogen bonds essentially disappear positive of the pzc, while hydrogen donating ones persist even very negative of the pzc because the lone pairs on the oxygen atom interact more strongly with the Au surface than the hydrogen atoms of water. This is consistent with the fact that negative of the pzc we observe a low-frequency tail of the *ν*_OD_ and *ν*_OH_, but no band at a frequency low enough as to be assigned to a hydrogen bond between water and the electrode surface. It is also consistent with the exchange of electronic density between water molecules and the electrode surface at the pzc (and, expectedly, positive of the pzc) recently unveiled by us,^27^ which has been suggested as being behind a negative component of the Helmholtz capacitance at the Pt(111)-water interface.^44^

The similar shape of the *ν*_OD_ and *ν*_OH_ bands on both sides of the pzc also suggests that reorientation occurs without significant disruption of the hydrogen bond network of interfacial water. The results of our simulations also support the presence of a hydrogen-bond network which is not disrupted by the potential-induced reorientation of the interfacial water molecules. The population of molecules with one of their O–H bonds parallel to the electrode surface remains nearly constant at all the simulated potentials, as does the population with their dipole moment perpendicular to the surface normal. When considered together, and when looking at the interfacial water layer as a whole rather than at individual water molecules, this suggests the presence of a stable backbone of hydrogen bonds parallel to the electrode surface that acts as an axis around which the potential-induced reorientation of the water dipoles occurs. While in acidic media hydrogen is produced by reduction of H^+^, in alkaline media the dissociation of H_2_O is required and is considered to be the rate-determining step, which also involves the transport of OH^−^ from the electrode surface to the bulk of the electrolyte.^47^ Based on this, it has been argued that promoting the reorientation of water with hydrogen pointing towards the electrode surface might be a way to increase the kinetics of the HER in alkaline media.^48^ However, this suggestion misses the point that in alkaline media the HER occurs at potentials negative of the pzfc in most, if not all, electrocatalysts, *i.e.*, under conditions in which, as we have shown, H_2_O is already oriented H-down. Alternatively, it has been suggested that the slower HER kinetics in alkaline media is due to a higher reorganisation energy resulting from the rigid water structure negative of the pzfc, which negatively affects the transport of OH^−^ away from the electrode through the double layer.^49,50^ Our conclusion that there exists a stable backbone of hydrogen bonds parallel to the electrode surface and that a considerable negative charge density on the electrode is needed to start breaking that backbone adds support to this latter hypothesis.

## Materials and methods

### ATR-SEIRA spectra

ATR-SEIRA spectra were acquired with a Nicolet iS50R spectrometer equipped with a liquid nitrogen-cooled MCT detector and using a homemade ATR accessory. The ATR element was a Si prism bevelled at 60° (Crystan). This was plated with gold using an electroless deposition method described elsewhere^51^ and the resulting thin gold film was used as the working electrode. Connection to the potentiostat was made by means of spring-loaded gold pins which were held in place by a homemade 3D printed piece which also served to hold the cell in position (see Fig. S4†). Spectra consisting of the coaddition of 100 interferograms were recorded during the positive cycle of a cyclic voltammogram at a scan rate of 5 mV s^−1^. Experiments were conducted at room temperature, approximately 21 °C, and normal atmospheric pressure. Time taken to acquire an individual spectrum at a resolution of 4 cm^−1^ was 17.3 s. Thus, the potential recorded for each spectrum is the average over the time of acquisition with one spectrum spanning 86 mV. Prior to conducting any experiment, the film was electrochemically annealed by cycling between 0.1 and 1.2 V at 50 mV s^−1^ for 30 min.

### Absorbance ATR spectra of bulk H_2_O : D_2_O mixtures

ATR absorption spectra of the bulk of 0.1 M HClO_4_ solutions in H_2_O : D_2_O 3 : 1 and 1 : 3 mixtures were recorded with the same spectrometer using a spectral resolution of 4 cm^−1^, after placing a drop of the electrolyte mixture on an uncoated prism using the spectrum of an uncoated and dry Si prism as background. Each spectrum is the result of coadding 16 interferograms, while 64 interferograms were accumulated for the background.

### Chemicals and reagents

The spectroelectrochemical cell was cleaned before use with piranha solution and then rinsed thoroughly with copious amounts of ultrapure water (18.2 MΩ cm) supplied by an Elga Purelab Chorus water-purification system. D_2_O, 99.9% D-atom, was obtained from Sigma Aldrich and used without further purification. Once filled with the electrolyte, the cell was deaerated with either nitrogen or argon (BOC) for a minimum of 10 minutes before measurements. The counter electrode was a gold wire, 99.999% purity metals basis (Alfa-Aesar) which was flame annealed to red heat before use. The potentiostat was an Emstat 3 Blue (Palmsense). The electrolyte was 0.1 M HClO_4_, prepared by diluting 70% HClO_4_, Emsure grade, purchased from Sigma Aldrich, unless otherwise stated.

Analytical grade NaAuCl_4_ (99%), Na_2_SO_3_ (98%), Na_2_S_2_O_3_·5H_2_O (99%), NH_4_Cl (99.5%) and HF (48%), all purchased from Sigma Aldrich, were used without further purification to prepare the gold-plating baths.

### Computational models

The Au(111) surface was modelled by 6 × 6 periodic slabs with 4 atomic layers. The length of the vacuum region between the lower and upper interfaces is ∼20 Å, and the size of the slab model is 17.615 × 17.615 × 28.191 Å^3^. The Au(111)–water interfaces were constructed by fully filling the vacuum region with water molecules, and the density of water in the bulk region was kept ∼1 g cm^−3^. The electric double layers (EDLs) at Au(111)–water interfaces were simulated by inserting ions near the Au surface. In our EDL model, the surface charge density can be controlled by varying the number of ions included. This method has been successfully applied to the oxide–water interface and metal–water interface.^33,44,52^ Based on this, we constructed four kinds of EDL models: inserting 4ClO_4_^−^, 8ClO_4_^−^, 4H^+^ and 8H^+^ in the upper and lower interfaces, respectively. Adding negative ions will raise the electrode potential and conversely positive ions will lower the electrode potential. By using the computational SHE method,^27^ the electrode potentials can be calculated. The electrode potential for the models containing no ion, 4ClO_4_^−^, 8ClO_4_^−^, 4H^+^ and 8H^+^ in the lower and upper interfaces are 0.35, 0.54, 0.95, −0.06 and −0.59 V, respectively. The calculated pzc differs from the value previously reported in ref. 27 by ∼0.15 V due to the uncertainty of ∼0.1 V in short statistical time.^27^ Since we are only concerned with the effect of the relative change in the electrode potential on the structure, we expect that the difference can be essentially cancelled out in the different models.

### Computational setup

All the calculations in this work were performed with CP2K.^53^ The DFT implemented in CP2K is based on a hybrid Gaussian plane wave (GPW) scheme that the orbitals are described by an atom-centred Gaussian-type basis set, and an auxiliary plane wave basis set is used to re–expand the electron density in the reciprocal space. The 1s electrons of H, the 2s, 2p electrons of O, the 3s, 3p electrons of Cl and the 5d, 6s electrons of Au were treated as valence, and the rest core electrons were represented by Goedecker–Teter–Hutter (GTH) pseudopotentials.^54,55^ The Gaussian basis set was double-ζ with one set of polarization functions (DZVP-MOLOPT-SR-GTH),^56^ and the energy cutoff for density expansion was set to 400 Ry. The Perdew–Burke–Ernzerhof (PBE) functional was used to describe the exchange-correlation energies,^57^ and the dispersion correction was applied in all calculations using Grimme D3 method.^58^

The second-generation Car–Parrinello molecular dynamics (SGCPMD)^59,60^ was used in all AIMD simulations. SGCPMD has been shown to be feasible for the simulation of different kinds of metal–water interfaces.^61,62^ In SGCPMD simulations, canonical ensemble (NVT) conditions were imposed by the Langevin thermostat and the temperature was set to 330 K. To control the temperature, two additional parameters are needed to set. One is *γ*_L_, the Langevin friction coefficient, and another is *γ*_D_, the intrinsic friction coefficient to control the dissipation. *γ*_L_ was set to 0.001 fs^−1^ and *γ*_D_ were 5 × 10^−5^ fs^−1^ for Au, 2.2 × 10^−4^ fs^−1^ for H_2_O and ions, respectively. The MD time step was set to 0.5 fs. For each simulation, an initial ∼5 ps of MD trajectory was used to equilibrate the system, and then followed by a production period of ∼10 ps. Due to the large size of the cells, only the *Γ* point in the reciprocal space was used for all simulations.

## Conclusions

Using Au as a model surface and combining potential-dependent vibrational spectra obtained using *in situ* ATR-SEIRAS with *ab initio* molecular dynamics at, as well as negative of and positive of, the pzc, we have provided deeper insight into the structure of water at electrode–electrolyte interfaces and its potential-induced reorientation.

We have shown that, in D_2_O : H_2_O mixtures, the interface is enriched in H atoms, due to the slightly larger dipole moment of D_2_O. Regarding the structure and hydrogen-bond network of interfacial water, the most important conclusions of our work are that (i) at room temperature there is no ice-like first layer of interfacial water at any potential, (ii) interfacial water reorients around a stable backbone of hydrogen bonds parallel to the electrode surface, (iii) a relatively low positive charge density on the electrode surface of 11.9 μC cm^−2^ is enough to orient nearly all surface water molecules with both oxygen lone pairs pointing towards the electrode and (iv) even a relatively high negative charge density on the electrode of −23.8 μC cm^−2^ is not enough to force water molecules to orient with their two hydrogen atoms pointing towards the electrode. At positive charge densities, interfacial water molecules form exclusively, or nearly exclusively, hydrogen-donating hydrogen bonds with water molecules on the electrolyte side of the double layer. At negative charge densities, however, the orientation is not strong enough as to make the population of hydrogen-donating water molecules vanish, and in fact the population of hydrogen-accepting water molecules is larger than that of hydrogen donors only at considerably negative charge densities. This can be attributed to the weak interaction of the hydrogen atoms of interfacial water molecules with the Au surface, as opposed to the stronger interaction of the surface with the lone pairs on the oxygen atoms. The larger number of hydrogen bonds per water molecule negative of the pzc than at positive charge densities probably impairs a higher rigidity to the interfacial water network, which could explain why the kinetics of the hydrogen evolution reaction (HER) on Pt and other electrodes are slower in alkaline than in acidic electrolytes.^47,49,63^

We expect our work to stimulate the deployment of H_2_O : D_2_O mixtures for vibrational studies of the structure and dynamics of water in the electrical double layer, a strategy that has proven extremely useful when applied to bulk water and water–air interfaces^2,4–7^ but which, to the best of our knowledge, had never been applied to electrode–electrolyte interfaces.

## Author contributions

AC conceived the idea and conceptualised the project, designed and supervised the experimental work, and wrote the manuscript. PG and AB performed all the experimental work and data compiling. XD and JL performed all the computational work. JC supervised the computational work. All authors contributed to revising the original manuscript.

## Conflicts of interest

There are no conflicts to declare.

## Supplementary Material

SC-015-D4SC04766D-s001

## Data Availability

The datasets generated and/or analysed during the current study are available from the corresponding author on reasonable request.

## References

[cit1] Soper A. K., Benmore C. J. (2008). Phys. Rev. Lett..

[cit2] Auer B. M., Skinner J. L. (2008). J. Chem. Phys..

[cit3] Kananenka A. A., Skinner J. L. (2018). J. Chem. Phys..

[cit4] Auer B. M., Skinner J. L. (2009). Chem. Phys. Lett..

[cit5] Schaefer J., Backus E. H. G., Nagata Y., Bonn M. (2016). J. Phys. Chem. Lett..

[cit6] Sovago M., Campen R. K., Wurpel G. W. H., Müller M., Bakker H. J., Bonn M. (2008). Phys. Rev. Lett..

[cit7] Seki T., Chiang K. Y., Yu C. C., Yu X., Okuno M., Hunger J., Nagata Y., Bonn M. (2020). J. Phys. Chem. Lett..

[cit8] Scherer J. R. , in Infrared and Raman Spectroscopy, ed. R. J. H. Clark and R. E. Hester, Heyden, Philadelphia, 1978, vol. 5

[cit9] Ataka K., Yotsuyanagi T., Osawa M. (1996). J. Phys. Chem..

[cit10] Shoesmith D. W., Lee W. (1976). Can. J. Chem..

[cit11] Jancso G., Van Hook W. A. (1974). Chem. Rev..

[cit12] Wolfsberg M. A. X. (1969). J. Chem. Phys..

[cit13] Hulston J. R. (1969). J. Chem. Phys..

[cit14] Friedman L., Shines V. J. (1966). J. Chem. Phys..

[cit15] Max J. J., Chapados C. (2002). J. Chem. Phys..

[cit16] Asbury J. B., Steinel T., Kwak K., Corcelli S. A., Lawrence C. P., Skinner J. L., Fayer M. D. (2004). J. Chem. Phys..

[cit17] Nienhuys H. K., Woutersen S., Van Santen R. A., Bakker H. J. (1999). J. Chem. Phys..

[cit18] Liang C., Rayabharam A., Aluru N. R. (2023). J. Phys. Chem. B.

[cit19] Clough S. A., Beers Y., Klein G. P., Rothman L. S. (1973). J. Chem. Phys..

[cit20] Jones G., Ray W. A. (1937). J. Chem. Phys..

[cit21] Stålgren J. J. R., Boschkova K., Ericsson J. C., Frank C. W., Knoll W., Satija S., Toney M. F. (2007). Langmuir.

[cit22] Pliskin W. A. (1977). J. Vac. Sci. Technol..

[cit23] Bockris J. O., Muller K. (1963). Proc. R. Soc. London, Ser. A.

[cit24] Damaskin B. B., Frumkin A. N. (1974). Electrochim. Acta.

[cit25] Parsons R. (1975). J. Electroanal. Chem..

[cit26] Fawcett W. R., Levine S., deNobriga R. M., McDonald A. C. (1980). J. Electroanal. Chem..

[cit27] Le J., Iannuzzi M., Cuesta A., Cheng J. (2017). Phys. Rev. Lett..

[cit28] Le J., Fan Q., Perez-Martinez L., Cuesta A., Cheng J. (2018). Phys. Chem. Chem. Phys..

[cit29] Le J., Cuesta A., Cheng J. (2018). J. Electroanal. Chem..

[cit30] Le J.-B., Cheng J. (2020). Curr. Opin. Electrochem..

[cit31] Kolb D. M. (1996). Prog. Surf. Sci..

[cit32] Thiel P. A., Madey T. E. (1987). Surf. Sci. Rep..

[cit33] Li C.-Y., Le J.-B., Wang Y.-H., Chen S., Yang Z.-L., Li J.-F., Cheng J., Tian Z.-Q. (2019). Nat. Mater..

[cit34] Ohno K., Okimura M., Akai N., Katsumoto Y. (2005). Phys. Chem. Chem. Phys..

[cit35] Walrafen G. E. (1970). J. Chem. Phys..

[cit36] Walrafen G. E. (1971). J. Chem. Phys..

[cit37] Mohandas N., Bawari S., Shibuya J. J. T., Ghosh S., Mondal J., Narayanan T. N., Cuesta A. (2024). Chem. Sci..

[cit38] Cheng J., Sprik M. (2012). Phys. Chem. Chem. Phys..

[cit39] Cheng J., Liu X., VandeVondele J., Sulpizi M., Sprik M. (2014). Acc. Chem. Res..

[cit40] Hussain G., Pérez-Martínez L., Le J.-B., Papasizza M., Cabello G., Cheng J., Cuesta A. (2019). Electrochim. Acta.

[cit41] Cicero G., Grossman J. C., Schwegler E., Gygi F., Galli G. (2008). J. Am. Chem. Soc..

[cit42] Nadler R., Sanz J. F. (2012). J. Chem. Phys..

[cit43] Bellarosa L., García-Muelas R., Revilla-López G., López N. (2016). ACS Cent. Sci..

[cit44] Le J. B., Fan Q. Y., Li J. Q., Cheng J. (2020). Sci. Adv..

[cit45] Toney M. F., Howard J. N., Richer J., Borges G. L., Gordon J. G., Melroy O. R., Wiesler D. G., Yee D., Sorensen L. B. (1994). Nature.

[cit46] Toney M. F., Howard J. N., Richer J., Borges G. L., Gordon J. G., Melroy O. R., Wiesler D. G., Yee D., Sorensen L. B. (1995). Surf. Sci..

[cit47] Dubouis N., Grimaud A. (2019). Chem. Sci..

[cit48] Cai C., Liu K., Zhang L., Li F., Tan Y., Li P., Wang Y., Wang M., Feng Z., Motta Meira D., Qu W., Stefancu A., Li W., Li H., Fu J., Wang H., Zhang D., Cortés E., Liu M. (2023). Angew. Chem., Int. Ed..

[cit49] Ledezma-Yanez I., Wallace W. D. Z., Sebastián-Pascual P., Climent V., Feliu J. M., Koper M. T. M. (2017). Nat. Energy.

[cit50] Sarabia F. J., Sebastián-Pascual P., Koper M. T. M., Climent V., Feliu J. M. (2019). ACS Appl. Mater. Interfaces.

[cit51] Osawa M., Ataka K., Yoshii K., Yotsuyanagi T. (1993). J. Electron Spectrosc. Relat. Phenom..

[cit52] Cheng J., Sprik M. (2014). J. Phys.: Condens. Matter.

[cit53] Kühne T. D., Iannuzzi M., Del Ben M., Rybkin V. V., Seewald P., Stein F., Laino T., Khaliullin R. Z., Schütt O., Schiffmann F., Golze D., Wilhelm J., Chulkov S., Bani-Hashemian M. H., Weber V., Borštnik U., Taillefumier M., Jakobovits A. S., Lazzaro A., Pabst H., Müller T., Schade R., Guidon M., Andermatt S., Holmberg N., Schenter G. K., Hehn A., Bussy A., Belleflamme F., Tabacchi G., Glöβ A., Lass M., Bethune I., Mundy C. J., Plessl C., Watkins M., VandeVondele J., Krack M., Hutter J. (2020). J. Chem. Phys..

[cit54] Hartwigsen C., Goedecker S., Hutter J. (1998). Phys. Rev. B.

[cit55] Goedecker S., Teter M. (1996). Phys. Rev. B.

[cit56] VandeVondele J., Hutter J. (2007). J. Chem. Phys..

[cit57] Perdew J. P., Burke K., Ernzerhof M. (1996). Phys. Rev. Lett..

[cit58] Grimme S., Antony J., Ehrlich S., Krieg H. (2010). J. Chem. Phys..

[cit59] Kühne T. D. (2014). Wiley Interdiscip. Rev.: Comput. Mol. Sci..

[cit60] Kühne T. D., Krack M., Mohamed F. R., Parrinello M. (2007). Phys. Rev. Lett..

[cit61] Li X. Y., Chen A., Yang X. H., Zhu J. X., Le J. B., Cheng J. (2021). J. Phys. Chem. Lett..

[cit62] Lan J., Hutter J., Iannuzzi M. (2018). J. Phys. Chem. C.

[cit63] Rheinländer P. J., Herranz J., Durst J., Gasteiger H. A. (2014). J. Electrochem. Soc..

