# Supplementary material for: Water at electrode–electrolyte interfaces: combining HOD vibrational spectra with ab initio-molecular dynamics simulations
Source: Chem Sci. 2024 Sep 23;15(42):17469–80. doi: 10.1039/d4sc04766d (PMC11447744; doi:10.1039/d4sc04766d)
Supplement: SC-015-D4SC04766D-s001 [file SC-015-D4SC04766D-s001.pdf]

Supplementary Information (SI) for Chemical Science.
This journal is © The Royal Society of Chemistry 2024

Supplementary Information

Water at electrode-electrolyte interfaces: combining HOD vibrational spectra with ab initio-molecular dynamics simulations

Pavithra Gunasekaran[1], Xianglong Du[2], Andrew Burley[1], Jiabo Le[3], Jun Cheng[2] and Angel Cuesta[1,4,*]

1. *Advanced Centre for Energy and Sustainability (ACES), School of Natural and Computing Sciences, University of Aberdeen, AB24 3UE Aberdeen, Scotland, UK*
2. *State Key Laboratory of Physical Chemistry of Solid Surfaces, iChem, College of Chemistry and Chemical Engineering, Xiamen University, Xiamen 361005, China*
3. *Ningbo Institute of Materials Technology and Engineering, Chinese Academy of Sciences, Ningbo, Zhejiang 315201, China*
4. *Centre for Energy Transition, University of Aberdeen, AB24 3FX, Aberdeen, Scotland, UK*

*Corresponding author: angel.cuestaciscar@abdn.ac.uk

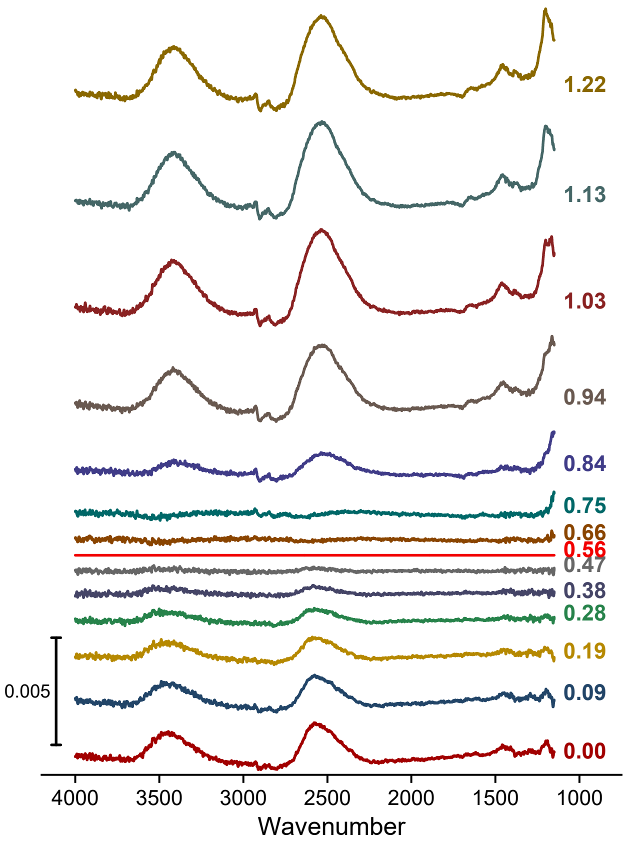

Figure S1. ATR-SEIRA spectral series between 0 and 1.2 V of the interface between a thin-film Au electrode deposited on Si and a 0.1 M H_2SO_4 solution in H_2O:D_2O 1:3.

Calculation of the electrode potential in the SHE in ab-initio molecular dynamics simulations

The electrode potential in the SHE scale, U_{SHE}, is calculated as:

$$eU_{SHE} = -E_F^{(i)} - e\phi_{wat}^{(i)} + \Delta_{DP}A_{H_3O^+}^{(w)} - \mu_{H^+}^{g,o} - \Delta E_{ZP} \quad (1)$$

Where e is the elementary charge, $E_F^{(i)}$ is the Fermi energy at the metal water interface, $\phi_{wat}^{(i)}$ is the average electrostatic potential of the water phase, computed under periodic boundary conditions (PBCs) in the same interface model, $\Delta_{DP}A_{H_3O^+}^{(w)}$ is the deprotonation free energy of H_3O^+(aq) computed under PBCs in a simulation box of pure water, $\mu_{H^+}^{g,o}$ is the standard chemical potential of gas phase proton and ΔE_{ZP} is a correction for the zero-point energy of the O-H bond in H_3O^+(aq). $\mu_{H^+}^{g,o}$ and ΔE_{ZP} are known constants of values 15.81 and 0.35 eV, respectively.[1] For $\Delta_{DP}A_{H_3O^+}^{(w)}$, the recommended value of 15.35 eV[2–4] is used. The values of $E_F^{(i)}$ and $e\phi_{wat}^{(i)}$ depend on the numbers of ions included in the simulation box (and therefore on the electrode charge density) and are obtained by averaging over an approximately 10 ps DFTMD trajectory. Fig. S2 shows DFTMD trajectories when there are no ions in the simulation cell (pzc, 0.35 V vs. SHE), when the simulation cell contains 4 and 8 ClO_4^- ions, respectively (σ_{Au} = 11.9 and 23.8 μC cm^{-2}, respectively, and U_{SHE} = 0.54 and 0.95 V, respectively) and when the simulation cells contains 4 and 8 H^+, respectively (σ_{Au} = -11.9 and -23.8 μC cm^{-2}, respectively, and U_{SHE} = -0.06 and -0.54 V, respectively). The difference between the calculated pzc of Au(111) reported here (0.35 V vs. SHE) and that recently reported by us (0.5 V vs. SHE[4]) stems from the use here of the SGCPMD AIMD protocol, whereas BOMD was used in reference [4]. SGCPMD is faster, but temperature control is more difficult. The finally simulated temperature is around 360 K, which is different from 330 K in our previous work. This different temperature is the main reason for the 0.15 V difference in the pzc. We have nonetheless checked that the high er temperature does not affect the effect of potential on the structures and that, therefore, the calculations can be compared with the experimental results.

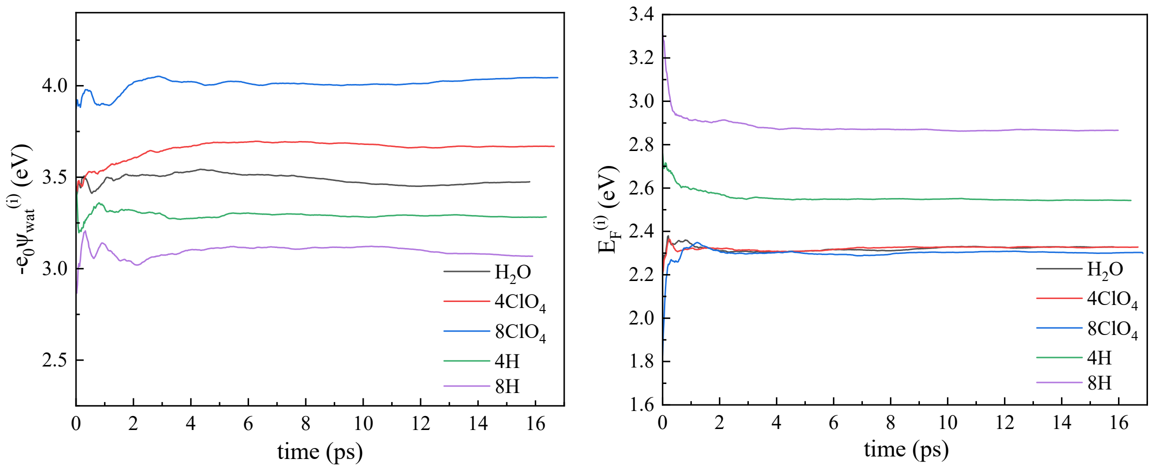

Figure S2. DFT-MD trajectories from which the average value of the electrostatic potential of bulk water (left) and the average Fermi level of the gold electrode (right) were calculated for the simulation cells containing only H_2O (black lines), 4 ClO_4^- (red), 8 ClO_4^- (blue), 4 H^+ (green) and 8 H^+

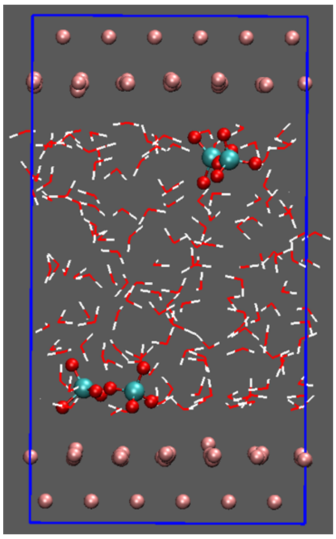

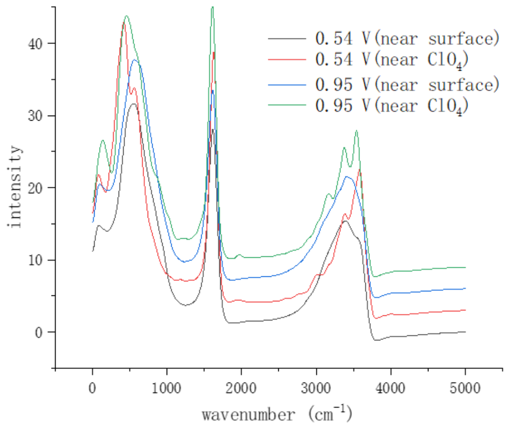

Figure S3. (a) Simulation cell used to calculate the VDOS of a water molecule in the solvation shell of ClO_4^-. **(b)** VDOS of an interfacial water molecule at 0.54 V (black line), of a water molecule in the solvation shell of interfacial ClO_4^- at 0.54 V (red line), of an interfacial water molecule at 0.95 V (blue line) and of a water molecule in the solvation shell of interfacial ClO_4^- at 0.95 V (green line). Only the VDOS of a water molecule in the solvation shell of interfacial ClO_4^- at 0.54 and 0.95 show maxima at ca. 3570 cm^{-1}. All potentials in the SHE scale.

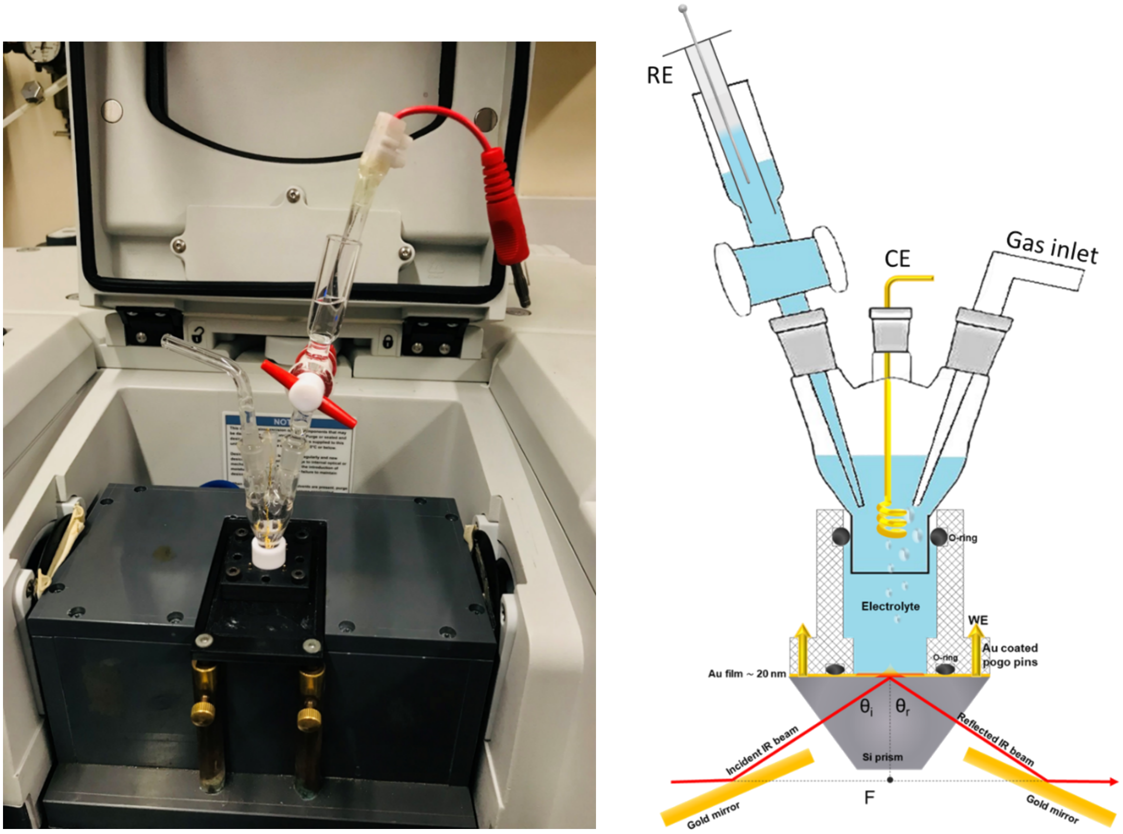

Figure S4. Photograph (left) and schematic diagram (right) of our spectroelectrochemical cell. The glass body of the cell fits into a Teflon piece which is held in place by a 3D printed resin piece screwed to the prism holder. Two O-rings are used to seal the space between the glass body of the cell and the cylindrical part of the teflon piece and to delimit the area of the gold film in contact with the electrolyte, respectively. Electric contact with the gold film is made via four spring-loaded gold pins at the four corners of the 3D printed resin piece screwed that holds the Teflon piece in place.

References

1. Cheng, J., Sulpizi, M. & Sprik, M. Redox potentials and pKa for benzoquinone from density functional theory based molecular dynamics. *J Chem Phys* **131**, 154504 (2009).

2. Cheng, J., Liu, X., VandeVondele, J., Sulpizi, M. & Sprik, M. Redox Potentials and Acidity Constants from Density Functional Theory Based Molecular Dynamics. *Acc Chem Res* **47**, 3522–3529 (2014).

3. Cheng, J. & Vandevondele, J. Calculation of Electrochemical Energy Levels in Water Using the Random Phase Approximation and a Double Hybrid Functional. *Phys Rev Lett* **116**, 086402 (2016).

4. Le, J., Iannuzzi, M., Cuesta, A. & Cheng, J. Determining Potentials of Zero Charge of Metal Electrodes versus the Standard Hydrogen Electrode from Density-Functional-Theory-Based Molecular Dynamics. *Phys Rev Lett* **119**, 016801 (2017).
